# Transforming Inpatient Venous Leg Ulcer Management Through Early Specialist Tissue Viability Intervention

**DOI:** 10.1111/iwj.71006

**Published:** 2026-07-28

**Authors:** Fania Pagnamenta, Rebecca Harris, Heather Agar, Catherine Burn

**Affiliations:** ^1^ Northumbria University Newcastle upon Tyne UK; ^2^ The Newcastle Upon Tyne Hospitals NHS Foundation Trust Newcastle upon Tyne UK

**Keywords:** compression bandages, leg ulcer, nursing care, venous insufficiency, wound healing

## Abstract

Inpatient care for venous leg ulcers is often inconsistent, with delays in vascular assessment and compression therapy contributing to prolonged healing and fragmented treatment. This evaluation examined a front‐door Tissue Viability Nurse‐led service providing specialist assessment at hospital admission. A descriptive service improvement evaluation was conducted. From 2022, patients in the Assessment Ward admitted with leg wounds received Tissue Viability assessment within one working day, including Doppler‐based vascular evaluation, wound bed preparation, peri‐wound skin optimisation and early initiation of compression therapy. Compression bandaging became a core clinical skill for registered nurses, supported by a rationalised product formulary. This observational evaluation and qualitative review suggested that early specialist involvement supported the timely initiation of compression therapy, reduced variation in inpatient wound care and minimised duplicate referrals through clear Tissue Viability‐led care plans. Staff confidence in applying compression was perceived to improve, collaboration with vascular, dermatology and community services strengthened and patients informally reported clearer information and greater continuity of care, supporting a timelier approach to care delivery. Positioning Tissue Viability expertise at the Assessment Ward appeared to support improvements in the consistency and safety of care processes in leg ulcer management. This front‐door specialist model, rarely described in acute care literature, enhanced timeliness and coordination and provides a scalable approach to addressing international gaps in acute wound care pathways.

## Key Points

- Inpatient venous leg ulcer care is often characterised by delays in vascular assessment and inconsistent use of compression therapy.
- A front‐door Tissue Viability Nurse‐led model enabled specialist assessment within one working day of hospital admission.
- Early specialist intervention supported timely initiation of compression therapy, standardised care planning, and improved continuity across acute and community services.
- Workforce development and simplified compression pathways increased staff confidence and reduced variation in inpatient leg ulcer management.
- This acute‐care service model provides a scalable approach to improving the consistency, coordination, and timeliness of leg ulcer care.

## 1 Introduction

Wound care represents a substantial component of nursing practice, with some clinical areas dedicating more than half of their workload to the assessment and management of wounds [1]. Venous leg ulcers are among the most common chronic wounds in the UK, affecting an estimated 1.5% of the population and placing a significant financial burden on the NHS, with annual costs approaching £1 billion [2, 3]. Their prevalence increases with age, particularly among people over 80, making effective and timely intervention increasingly important for a growing older population. Despite their prevalence and impact, the management of venous leg ulcers in acute care remains highly variable. More than two‐thirds of NHS hospitals do not have a dedicated inpatient leg ulcer service and only a minority routinely provide compression therapy, the primary treatment for venous disease [4]. Organisational pressures, limited specialist availability and inconsistent pathways often lead to delays or omissions in compression initiation, contributing to prolonged healing, unnecessary variation in practice and poorer patient experience [5, 6].

Venous leg ulcers are chronic wounds characterised by repeated cycles of breakdown and delayed healing [7, 8]. Although most ulcers can heal within a year, a significant minority remain unhealed for several years and recurrence rates remain high [7, 8]. While care is predominantly delivered in the community, hospital admissions for unrelated conditions frequently interrupt established treatment plans. When communication between acute and community teams is limited, these disruptions can delay the re‐initiation of therapy and contribute to inconsistent practice and avoidable complications [9]. Compression therapy, the cornerstone of venous leg ulcer management, remains inconsistently provided in acute care settings due to organisational constraints such as staffing pressures, competing clinical demands, unclear referral pathways and variable access to compression products [5, 6]. Clinicians may also lack confidence or training in achieving safe, therapeutic compression levels [10]. Patient‐related factors, including discomfort, limited understanding of compression's importance and challenges maintaining therapy after discharge, further affect continuity of care [11, 12]. Consequently, many patients are discharged without appropriate compression, leaving community teams to restart therapy, often weeks later, prolonging healing times and reinforcing fragmented care pathways [13].

The burden of venous leg ulcers extends beyond clinical outcomes, contributing to pain, reduced mobility, diminished quality of life and substantial financial pressure, with annual NHS costs nearing £1 billion [2, 3]. Despite the known benefits of compression therapy, inpatient management continues to vary widely. Such variation reflects broader issues in service organisation, workforce capability and adherence to national guidance. Longstanding practice patterns also play a role: it remains common for community‐applied compression to be removed on admission and replaced with simple dressings or light bandages, despite strong evidence and national guidelines emphasising the need for therapeutic compression to address underlying venous hypertension [14, 15]. This entrenched approach leads to treatment delays, inconsistent practice and prolonged healing trajectories, highlighting the need for more systematic inpatient pathways [14, 16]. Introducing early specialist Tissue Viability involvement at the point of admission offers an opportunity to address these challenges by supporting consistent assessment, timely compression initiation and improved continuity between acute and community services [14, 15, 17].

This service improvement project describes the introduction of early Tissue Viability specialist involvement within the Assessment Ward of a large tertiary hospital in the North East of England. In this hospital, the Assessment Ward functions as the acute admission assessment area where patients are triaged and directed to the appropriate inpatient speciality. The aim was to deliver timely, standardised care for patients presenting with leg ulcers or lower‐limb skin concerns, supporting improvements in care processes and clinical management and enhancing the confidence and capability of the wider nursing workforce. The aims and objectives of this service evaluation are outlined below.

### 1.1 Aims and Objectives

The primary aim of this quality initiative was to enhance the early management of leg ulcers in the Assessment Ward through the integration of specialist Tissue Viability assessment and intervention at the point of admission.

The specific objectives were to describe the impact of early specialist involvement on the timeliness and consistency of compression therapy, explore its influence on generalist nursing practice and confidence in wound care, examine its role in supporting continuity of care between acute and community settings and consider its perceived contribution to reducing delays in the initiation of compression therapy.

## 2 Methods

### 2.1 Study Design and Governance

This work is a clinical service improvement evaluation conducted within routine care. Although it drew on the principles of the Plan–Do–Study–Act (PDSA) framework [18, 19], the model was applied as a single, structured cycle rather than as multiple iterative cycles typically associated with this approach. Its primary purpose was to assess feasibility, acceptability and process impact when embedding early Tissue Viability input at the point of admission, rather than to test a hypothesis under research conditions. As such, the activity was undertaken as service evaluation and quality improvement within organisational governance frameworks; no alteration to standard care was required beyond pathway optimisation, staff education and product rationalisation. Consistent with local policy for service evaluation, formal research ethics committee approval was not required. This evaluation was designed as a descriptive service improvement study focused on process, feasibility and perceived impact rather than the measurement of predefined clinical outcomes. The evaluation therefore focuses on service processes, clinical decision‐making and implementation within real‐world practice rather than hypothesis testing.

### 2.2 PDSA Structure and Learning

In this evaluation, the PDSA framework was used as a guiding structure rather than as a full iterative cycle methodology, helping to ensure clarity and avoid misinterpretation of the approach. Although informed by PDSA principles, the service redesign was implemented as a single comprehensive cycle rather than multiple iterative cycles. All key components—front‐door wound screening, rapid specialist assessment, streamlined vascular evaluation, product rationalisation and a competency‐based training package—were introduced simultaneously as part of a coordinated pathway redesign. The ‘study’ phase focused on monitoring feasibility, acceptability and workflow impact during real‐time delivery, with learning centred on the importance of early clinical assessment, simplified compression options and embedding clear tissue‐viability‐led care plans within the electronic record. These insights informed minor adjustments to day‐to‐day practice but did not constitute separate cycles, meaning the model represents a whole‐system change implemented in one strategic phase rather than an incremental sequence of tests. The use of PDSA terminology therefore reflects the conceptual framework underpinning the evaluation rather than a traditional multi‐cycle improvement process.

### 2.3 Why This Evaluation Contributes New Insight

Although the effectiveness of compression therapy and structured leg‐ulcer pathways is well established, implementation in inpatient acute settings remains inconsistent. The novel contribution of this evaluation is to describe and assess a front‐door TV‐led model, daily screening, rapid vascular assessment, early wound bed preparation and timely, protocolised compression, implemented at the Assessment Ward. This ‘front‐door early specialist’ configuration is under‐reported compared with community‐based or outpatient pathway redesigns. By detailing the operational components (early clinical assessment, assessment timing, electronic documentation, product formulary and competency framework) and the observed service‐level effects (reduced duplicate referrals, earlier initiation of compression, strengthened cross‐boundary communication), this paper provides a replicable blueprint that other providers can adapt within their own contexts.

### 2.4 Screening and Referral

All emergency admissions were subjected to wound screening (undertaken by the Assessment Ward's nursing and medical staff), with patients presenting with lower‐limb wounds or clinically significant dermatological changes referred to the Tissue Viability team. This approach aligns with national guidance that underscores the importance of early wound identification and timely specialist involvement to optimise clinical outcomes [17].

### 2.5 Case Definition

Venous leg ulcers were identified based on clinical assessment, including anatomical location within the gaiter region, wound characteristics and vascular assessment findings. Doppler waveform analysis was used to confirm the presence of adequate arterial supply. Where diagnostic uncertainty existed, wounds were managed as mixed aetiology and compression therapy was modified accordingly in line with international guidance [16, 20].

### 2.6 Assessment

A comprehensive assessment was conducted by a Tissue Viability Nurse Specialist within one working day, incorporating direct visual examination of the wound following removal of existing dressings and the use of medical photography to facilitate precise documentation and longitudinal evaluation of wound progression. Vascular evaluation predominantly utilised hand‐held Doppler ultrasonography to characterise arterial waveforms, with ankle–brachial pressure index used selectively and duplex arterial imaging performed when clinically warranted. A streamlined assessment incorporating clinical indicators such as pedal pulses, limb temperature, capillary refill and tissue perfusion supported this process. The assessment also involved a broad evaluation of the patient's mobility, level of discomfort, skin condition and relevant health conditions, in keeping with patient‐centred frameworks that recognise the wide range of factors that can influence wound healing [21]. Crucially, prioritising Doppler waveform assessment over routine ABPI enabled much earlier initiation of compression therapy, allowing treatment to begin promptly, typically at a reduced level before progressing to full compression within a few days once no treatment‐related complications were observed, thereby reducing delays that traditionally hinder effective inpatient leg‐ulcer management.

Where required, ankle–brachial pressure index (ABPI) was used to support clinical decision‐making, with values ≥ 0.8 considered indicative of suitability for full compression and values between 0.5 and 0.8 guiding the use of reduced or modified compression, in accordance with established clinical guidance [16, 20].

### 2.7 Intervention

Therapeutic interventions were commenced immediately following the completion of the initial clinical assessment. Wound bed preparation and peri‐wound integument optimisation were undertaken in accordance with international consensus‐based wound‐care pathways that advocate a structured, evidence‐driven approach to establishing a physiologically favourable healing environment [22]. Sharp conservative debridement was performed where clinically indicated, consistent with guidance emphasising the timely removal of devitalised or non‐viable tissue to reduce bioburden and facilitate progression to the reparative phase [17]. Compression therapy was prescribed and initiated in line with vascular assessment findings, ensuring adherence to established clinical standards for the management of lower‐limb venous and mixed‐aetiology wounds [17]. Individualised management plans were generated electronically and disseminated to both inpatient clinical teams and community nursing services to support seamless continuity of care in accordance with NHS governance and local policy requirements.

### 2.8 Procurement Streamlining

Compression therapy was delivered using a standardised two‐layer system. Light compression, delivering approximately 20–30 mmHg at the ankle, was used where clinical caution was required, including in patients with borderline vascular status or limited tolerance. This was typically progressed to full compression, delivering approximately 40 mmHg at the ankle, once safety and tolerance had been established. Both ‘dry’ systems and zinc‐based (‘wet’) systems were used, selected according to wound characteristics, exudate level and skin condition, in line with best practice guidance [20].

### 2.9 Workforce Development

Assessment of vascular status represents the most complex aspect of compression therapy, as it is an essential prerequisite for ensuring treatment safety [20]. For this reason, all vascular assessments were consistently performed by the Tissue Viability Nurse Specialists. Once the patient's vascular status had been confirmed, generalist nursing staff were supported through targeted training in the application of modern compression systems, which are designed to be straightforward and user‐friendly. As the service evolved, compression bandaging became formally recognised within the organisation as a core wound‐care competency. To support this, an online educational package was introduced, incorporating e‐learning modules, demonstration videos and a self‐assessment for competency. This approach aligns with educational strategies described in the literature, which highlight the value of blended digital learning to enhance proficiency in compression therapy [23].

### 2.10 Tissue Viability Team Capacity and Activity

The in‐patient Tissue Viability service manages more than 7000 referrals each year, with approximately 22.5% (*n* = 1375) relating to leg ulcers. The service is delivered by a team comprising one Clinical Academic Nurse Consultant (0.45 WTE clinical), three Tissue Viability Nurse Specialists (2.1 WTE) and one Associate Practitioner (1 WTE), working across two hospital sites located 1.2 miles apart.

### 2.11 Use of AI Writing Assistance

Microsoft 365 Copilot (enterprise version integrated within Microsoft Word, 2024 release) was employed during manuscript preparation solely for grammar and spelling checks and minor phrasing improvements. The first author independently reviewed, verified and revised all material and accepts full responsibility for the final content. No analytical procedures or scientific interpretations were generated using artificial intelligence.

## 3 Results

Early specialist assessment appeared to support a reduction in repeat ward referrals for the same patient and condition, with the introduction of clear wound care plans at the point of admission providing teams with the information needed to avoid unnecessary duplication, although this observation was not derived from a discrete quantitative metric. Multidisciplinary working also appeared to strengthen, particularly with vascular, dermatology and community nursing teams, supporting a more coordinated and seamless approach to wound care. Staff confidence was perceived to improve, especially in the safe delivery of compression therapy, reflecting the impact of focused training and sustained clinical support. Informal patient feedback was positive, with reports of improved consistency, clarity and overall quality of care. Over time, these developments were associated with a broader cultural shift, with leg ulcer care becoming more proactive, collaborative and embedded into routine practice.

Service activity data were analysed to provide contextual insight into changes in referral patterns over time, as an indicator of service demand and pathway utilisation.

Although referral data relate to leg ulceration more broadly, venous aetiology was determined clinically by the Tissue Viability team as described above. Routine service datasets did not differentiate ulcer aetiology at the point of referral, which represents a limitation of this evaluation.

Analysis of referral activity (Table 1) to the inpatient Tissue Viability service from 2023 to 2025 shows significant shifts in both the volume of wound‐related demand and the routes through which patients accessed the service. Overall referrals to the Inpatient TV Team rose sharply by 42.9% between 2023 and 2024, before stabilising with a modest 3.7% reduction in 2025. Despite this slight decrease, activity remained considerably higher than in 2023, indicating sustained growth in clinical complexity, heightened organisational awareness of tissue viability issues and stronger adherence to referral processes. In contrast, referrals specifically relating to leg ulceration continued to increase year on year, rising by 17.9% from 2023 to 2024 and by a further 4.7% in 2025. This ongoing upward trend highlights the persistent burden of chronic wounds among the inpatient population.

**TABLE 1:** Number of referrals to the inpatient tissue viability team versus assessment ward.

	Number of referrals to inpatient TV team	Number of referrals for leg ulceration	Number of referrals from the assessment ward for all wounds	Number of referrals from the assessment ward for leg ulceration
2023	4641	1093	519	139
2024	6635	1289	621	167
2025	6387	1350	436	115

The most prominent shift relates to referral activity from the Assessment Ward. Although referrals from this area rose by 19.6% between 2023 and 2024, they declined markedly in 2025, with a 29.8% reduction across all wound types and a 31.1% decrease for leg ulceration specifically. Importantly, this drop may reflect pathway‐related changes rather than a genuine reduction in wound prevalence, although this cannot be confirmed from the available data. The data suggest a possible influence of operational adjustments within the admission process on where referrals were recorded, but this interpretation remains tentative without supporting operational metrics. In practice, Assessment Ward staff may initiate a referral, but by the time the Tissue Viability team attends, the patient has already moved, which may mean that referral locations sometimes reflected the ward where the patient was seen rather than where the referral originated, although this was not formally measured. This pattern could be consistent with a redistribution of referral sources rather than a reduction in tissue viability needs, but further data would be required to verify this explanation.

Taken together, the dataset illustrates that although demand for tissue viability expertise, especially in chronic leg ulceration, has grown steadily over the three‐year period, the mechanisms through which patients are referred have shifted. These findings highlight the importance of monitoring referral pathways alongside activity levels, as operational changes within acute care can directly affect service workload and visibility. Ongoing review will be essential to understand the drivers behind these patterns, maintain equitable and timely identification of wound care needs and inform strategic planning for future tissue viability provision.

These referral trends also offer insight into how pathway redesign influenced the timeliness and efficiency of care. Although referrals from the Assessment Ward decreased in 2025, this change may reflect pathway‐related operational factors rather than a true reduction in wound prevalence, particularly as total leg‐ulcer referrals continued to rise. The numerical patterns suggest a possible influence of faster patient flow and earlier specialist assessment on where referrals were recorded, but this interpretation is tentative, as the evaluation did not include operational timing data. It is also possible that some referrals were logged on the ward where the patient was ultimately seen rather than the ward that initiated the referral, although this was not formally measured. Overall, these trends could be consistent with a redistribution of referral sources associated with pathway redesign, but further data would be required to verify this explanation.

## 4 Discussion

Embedding Tissue Viability specialist input at the point of admission appears to support earlier initiation of key interventions such as vascular assessment, wound bed preparation, peri‐wound skin optimisation and timely compression therapy, all of which are central to effective venous leg ulcer management [2, 17, 24]. Although evidence shows that early compression contributes to improved healing outcomes [17, 24, 25], this evaluation did not measure clinical endpoints and the observed changes relate primarily to pathway processes.

The service appeared to help to address organisational barriers, including inconsistent staff confidence, limited access to compression systems and delays caused by ward transfers or unclear pathways [6, 10, 20]. By ensuring timely vascular assessment from specialist nurses and embedding clear wound care plans within the electronic record, the model appeared to reduce variation and improve clarity for wards and community teams, an approach that reflects national calls for stronger pathways and organisational support [15, 23].

Poor information transfer is a well‐recognised contributor to inconsistent therapy following discharge and delays in re‐initiating treatment after admission [9]. The organisation's electronic documentation system has strengthened Tissue Viability's ability to create clear, accessible wound care plans for ward staff to follow and has appeared to strengthen continuity of care between acute and community services.

Workforce development emerged as a critical enabler. Recognising compression application as a core organisational competency, supported by blended e‐learning and practical assessment, was associated with improved nursing confidence in an area where clinicians often report limited proficiency [10, 23]. Simplifying procurement to a smaller number of compression systems further supported consistent skill development and reduced unnecessary variation [15, 20].

Overall, the project suggested that early specialist involvement supports timely, safe and standardised wound care in acute settings. While the evaluation relied primarily on descriptive outcomes, improvements in consistency, staff confidence and multidisciplinary communication suggest that this model has the potential to reduce unwarranted variation and enhance inpatient leg ulcer management [6, 13]. Future work incorporating quantitative outcome measures, such as time to compression, healing trajectories, or patient‐reported outcomes, would strengthen the evidence base and allow more robust evaluation of impact [7, 24].

### 4.1 Interpretation and Implications for Practice

From a nursing practice perspective, the model suggests how early specialist involvement can help generalist ward teams deliver safe, timely wound care. By ensuring early vascular assessment, wound bed preparation, peri‐wound skin care and initiation of compression occur early in the admission process, the service supports care pathways aligned with best practice recommendations [17, 22]. The increased confidence among staff mirrors findings from educational research demonstrating the effectiveness of blended training approaches for improving compression skills [23].

### 4.2 Strengths of the Service Approach

Key strengths include consistent front‐door specialist screening, assessment protocols, structured workforce support and integrated electronic documentation. These elements are directly responsive to known organisational and clinical challenges, offering a replicable model for other acute settings [4, 6].

### 4.3 Global Relevance of the Model

The challenges addressed by this service, delayed initiation of compression, inconsistent vascular assessment, disrupted community treatment plans and variable staff confidence, are widely recognised across international wound‐care systems [10, 23]. Similar barriers to delivering timely, evidence‐based compression and maintaining continuity across care settings have been reported in multiple countries, reflecting system‐level issues rather than local anomalies [6, 20]. The core elements of this model, early specialist screening at admission, safety‐focused vascular assessment, simplified compression choices, documentation and targeted staff training, align with internationally endorsed principles for improving consistency, safety and continuity in venous leg ulcer care [15, 22]. Because these components are not dependent on specific organisational structures or funding models, the approach is adaptable to diverse healthcare contexts and offers a scalable solution to well‐documented global gaps in inpatient wound care.

### 4.4 Challenges and Limitations

The current evaluation relied primarily on descriptive outcomes and did not include predefined quantitative measures or formal statistical analysis. As such, findings should be interpreted as descriptive and hypothesis‐generating rather than definitive evidence of effectiveness. While observations such as earlier compression initiation and perceived improvements in staff confidence were noted, the absence of quantitative process or clinical outcome data limits the strength of the conclusions. This reflects a common challenge in service redesign projects, where operational pressures frequently constrain opportunities for comprehensive data collection [26].

To strengthen future evaluations, incorporating measures such as time to compression initiation, healing trajectories, recurrence rates and patient‐reported outcomes would provide more robust evidence of impact [7, 24]. Furthermore, findings are derived from a single tertiary centre and may not be generalisable to organisations with different staffing models, digital systems, or procurement pathways. Ongoing competence maintenance will also require regular education updates, particularly in the context of workforce turnover and rotation, a challenge well recognised across wound care services [6, 20].

### 4.5 Recommendations for Future Evaluation

While this evaluation provides a detailed descriptive account of service implementation and its perceived impact, the absence of prospective quantitative outcome measures represents an important limitation. Future work should incorporate routinely collected audit data to evaluate measurable indicators such as time from admission to vascular assessment or compression initiation [17], proportion of patients receiving compression prior to discharge, wound progression and healing rates and recurrence [8, 25].

In addition, balancing measures such as compression‐related complications or dressing change frequency [23], alongside experience measures including structured staff confidence scores and patient‐reported outcomes [6, 12], would provide a more comprehensive assessment of service impact. This approach would enable a more robust evaluation of both clinical effectiveness and patient experience and help determine the extent to which early intervention influences care delivery and outcomes.

## 5 Conclusion

Embedding Tissue Viability expertise within the Assessment Ward has created a foundation for long term service improvement by normalising early holistic vascular screening, promoting greater clinical accountability which has contributed to more consistent approaches to lower‐limb wound management by ensuring that interventions are initiated at the earliest opportunity. Early specialist assessment appeared to reduce the likelihood of delays commonly caused by ward transfers, variable staff confidence and inconsistent approaches to compression therapy. This model appeared to support nurses in delivering more consistent care, with perceived improvements in safety and effectiveness by combining structured education, simplified procurement processes and improved access to specialist support.

Establishing compression therapy as a core organisational skill was associated with improvements in perceived workforce capability and addressed long‐standing gaps in inpatient leg ulcer care. Integrating clear tissue viability documentation within the electronic record further supported continuity across acute and community pathways, helping patients receive consistent care regardless of clinical setting.

This shift from variable practice to a coordinated, pathway‐led approach illustrates the impact of thoughtful service redesign and strong clinical leadership. By aligning practice with contemporary evidence and national guidance, the initiative appears to have improved aspects of patient experience, supported more efficient service delivery and may have contributed to safer and more effective management of venous leg ulcers within the acute care environment. It highlights the potential for early Tissue Viability involvement to drive sustained improvements in outcomes and reduce unwarranted variation in wound care provision.

## Funding

The authors have nothing to report.

## Ethics Statement

This project was conducted as a service evaluation and quality‐improvement activity within organisational governance processes. In accordance with local policy and national guidance, formal research ethics committee approval was not required and individual patient consent was not necessary, as no interventions beyond usual care were introduced and no identifiable data were used.

## Conflicts of Interest

The authors declare no conflicts of interest.

## Data Availability

The data that support the findings of this study are available from the corresponding author upon reasonable request.
